# Synergism of the Combination of Traditional Antibiotics and Novel Phenolic Compounds against *Escherichia coli*

**DOI:** 10.3390/pathogens9100811

**Published:** 2020-10-03

**Authors:** Md. Akil Hossain, Hae-Chul Park, Sung-Won Park, Seung-Chun Park, Min-Goo Seo, Moon Her, JeongWoo Kang

**Affiliations:** 1 Veterinary Drugs & Biologics Division, Animal and Plant Quarantine Agency, Gimcheon-si 39660, Korea; mdakil_hossain@yahoo.com (M.A.H.); sungpark@korea.kr (H.-C.P.); pasawa@korea.kr (S.-W.P.); herm@mail.go.kr (M.H.); 2 Laboratory of Veterinary Pharmacokinetics and Pharmacodynamics, College of Veterinary Medicine, Kyungpook National University, Bukgu, Daegu 41566, Korea; parksch@knu.ac.kr; 3 Division of Vectors and Parasitic Diseases, Korea Centers for Diseases Control and Prevention, Cheongju 28159, Korea; koreasmg@korea.kr

**Keywords:** synergistic effect, antibacterial agents, bacterial pathogenicity, ampicillin, erythromycin

## Abstract

Pathogenic *Escherichia coli* (*E. coli*)-associated infections are becoming difficult to treat because of the rapid emergence of antibiotic-resistant strains. Novel approaches are required to prevent the progression of resistance and to extend the lifespan of existing antibiotics. This study was designed to improve the effectiveness of traditional antibiotics against *E. coli* using a combination of the gallic acid (GA), hamamelitannin, epicatechin gallate, epigallocatechin, and epicatechin. The fractional inhibitory concentration index (FICI) of each of the phenolic compound-antibiotic combinations against *E. coli* was ascertained. Considering the clinical significance and FICI, two combinations (hamamelitannin-erythromycin and GA-ampicillin) were evaluated for their impact on certain virulence factors of *E. coli*. Finally, the effects of hamamelitannin and GA on *Rattus norvegicus* (IEC-6) cell viability were investigated. The FICIs of the antibacterial combinations against *E. coli* were 0.281–1.008. The GA-ampicillin and hamamelitannin-erythromycin combinations more effectively prohibited the growth, biofilm viability, and swim and swarm motilities of *E. coli* than individual antibiotics. The concentration of hamamelitannin and GA required to reduce viability by 50% (IC_50_) in IEC-6 cells was 988.54 μM and 564.55 μM, correspondingly. GA-ampicillin and hamamelitannin-erythromycin may be potent combinations and promising candidates for eradicating pathogenic *E. coli* in humans and animals.

## 1. Introduction

*Escherichia coli* (*E. coli*) is a common, rod-shaped, gram-negative bacteria belonging to the *Enterobacteriaceae* family. Many *E. coli* strains in the intestinal tract of humans and animals are considered as commensal members of the microbiota [1]. However, some strains, including enteropathogenic *E. coli*, enteroinvasive *E. coli*, enteroaggregative *E. coli*, enterotoxigenic *E. coli*, and enterohemorrhagic *E. coli*, are highly pathogenic, and may initiate a wide spectrum of diseases ranging from self-limiting to life-threatening intestinal and extra-intestinal illnesses, such as enteritis, pyelonephritis, enterotoxaemia, mastitis, cystitis, meningitis, septicemia, and arthritis [1,2,3]. Antibiotics have long been considered the first line of defense to prevent pathogenic *E. coli*-associated infections. However, the treatment of pathogenic *E. coli* has become complicated by the rapid emergence and dissemination of antibiotic-resistant strains [2]. 

Infectious diseases associated with these antibiotic-resistant bacterial strains cause both direct and indirect losses to the livestock sector. Direct losses are associated with the escalation of livestock mortality and the reduction of livestock productivity; food insecurity, decreased market value, loss of trade, and cost of control are considered indirect losses [4]. Moreover, 75% of arising infectious-diseases in humans are known to have animal origins, suggesting that animal is a major reservoir of infections [5]. The transmission of antibiotic-resistant microorganisms (ARMs), including *E. coli*, between livestock and humans may occur easily, via contaminated water, food, waste, or any other environmental factor [6]. Infectious agents, such as antibiotic-resistant *E. coli* disseminated between animal and human are significant for public health, as well as for livestock economics [7]. As the currently used antibiotics have started to lose their effectiveness because of the occurrence of multidrug-resistant strains, novel approaches are required urgently to minimize the bacterial load in food-producing animals, which will eventually diminish the public health risks.

Combining antibacterial agents is one such novel and opportunistic strategy that may enhance the efficacies of currently available antibiotics in the inhibition of multidrug-resistant bacterial strains, such as *E. coli* [8]. The antimicrobial, antioxidant, and anticarcinogenic effects of phenolic compounds from natural sources have been reported [9,10]. In our previous studies, we found that *Nymphaea tetragona* 50% methanol extract (NTME), which contains a large proportion of phenolic compounds namely methyl gallate and pyrogallol, has synergistic antibacterial, virulence factor inhibition and anti-quorum sensing effects [11,12]. It was demonstrated that the biofilms of *Streptococcus mutans* could be efficiently inhibited by the treatment of the phenolic compound, gallic acid (GA) [13]. A GA derivative, methyl gallate, was described in our recent reports for its inhibitory effects on the adhesion, invasion, and intracellular survival of *Salmonella enterica* serovar *typhimurium*, and for its interfering effects on *Pseudomonas aeruginosa* quorum-sensing regulatory genes [14,15]. These properties of bacteria play a vital role in increasing antimicrobial resistance and pathogenicity [16].

GA derivatives contain many hydroxyl groups. Bacterial inhibition may occur through the large number of hydroxyl groups, owing to their ability to form ionic and protonic bonds and deactivate many biological proteins, for example, receptors, ion channels, enzymes, and carriers, by binding with them. Moreover, the molecular targets of microorganisms can be non-specifically affected by the phenolic groups of GA derivatives [17]. Based on the abovementioned properties of GA derivatives and our previous findings related to this group of compounds, we speculated that GA derivatives may have the potential to increase the efficacy of currently available antibiotics. Therefore, we designed the current research to investigate the anti-bacterial effects of GA, hamamelitannin (HAMA), epigallocatechin, epicatechin gallate, and epicatechin, individually and in combination with eight commercial antibiotics; ampicillin (AMP), amoxicillin, ceftiofur, penicillin G, cefotaxime, erythromycin (ERY), thiamphenicol, and marbofloxacin against *E. coli*. Furthermore, the impacts of these combinations of traditional antibiotics and phenolic compounds against certain pathogenic factors, such as motility and biofilm, were assessed. Lastly, the impacts of most potential phenolic compounds alone and combined with currently available antibiotics on cell viability were examined. A portion of this study has recently been published, where we reported the combination effects of traditional antibiotics and phenolic compounds in the eradication of *Salmonella enterica serovar* Typhimurium [18].

## 2. Results

### 2.1. Minimum Inhibitory Concentration of Phenolic Compounds and Antibiotics

The minimum inhibitory concentration (MIC) values of 10 currently available antibiotics (AMP, amoxicillin, ceftiofur, penicillin G, ERY, cefotaxime, thiamphenicol, norfloxacin, marbofloxacin, and florfenicol) and five phenolic compounds (GA, HAMA, epigallocatechin, epicatechin gallate, and epicatechin) against the quality control strain and the clinical isolates of *E. coli* were determined. The MIC values of the abovementioned antibiotics against the quality control strain of *E. coli* ranged from 0.125–128.00 µg/mL. Moreover, the MIC values of these antibiotics against *E. coli* field isolates ranged from 0.25 to >1024 µg/mL. However, the MIC value of penicillin G against the *E. coli* field isolate (V03-13-A03-002-009) was >1024 µg/mL. Increases of several folds in the MIC values of most of the tested antibiotics against *E. coli* field isolates were found in this study (Table 1). These results demonstrated that the studied clinical strains of *E. coli* have developed resistance to most of the tested antibiotics [19,20,21,22,23,24,25]. The MIC values of these tested phenolic compounds ranged from 32.00 µg/mL to 2048.00 µg/mL against the quality control strain of *E. coli*.

### 2.2. Antibacterial Effects of Combinations of Phenolic Compound and Antibiotic 

The combination effects of commercially available antibacterial agents and phenolic compounds against *E. coli* ATCC25922 were determined from the fractional inhibitory concentration index (FICI) values by performing checkerboard microdilution assays. The results of the combined activities of these commercial antibacterial agents and phenolic compounds are shown in Table 2. The GA-thiamphenicol, HAMA-ERY, and HAMA-thiamphenicol combinations represented synergistic antibacterial effects against this strain with their FICI values of 0.281, 0.375, and 0.50, respectively. Nevertheless, 17 combinations were found to exhibit additive antibacterial effects (FICI: 0.502–0.625) against this strain. Other combinations against this bacterial strain demonstrated indifferent antibacterial effects, with no evidence of antagonistic effects from any combination. In view of their clinical and commercial significance, two combinations (AMP-GA, and ERY-HAMA), which were found to have synergistic or additive effects, were chosen for more studies.

### 2.3. Effects of Phenolic Compound-Antibiotic Combinations on Concentration- and Time-Dependent Bacterial Inhibition Rate

**Figure 1:**
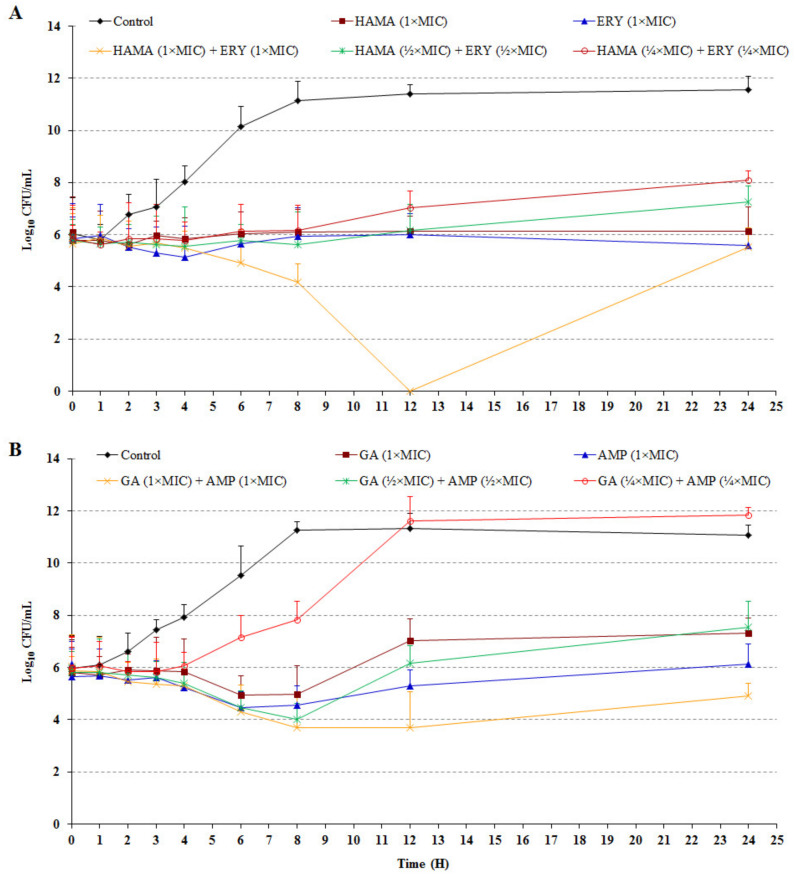
Time-kill curves of *Escherichia coli* ATCC25922 after treatment with (**A**) HAMA-ERY combination, and (**B**) GA-AMP combination. Data were analyzed by one-way analysis of variance with F-test. Data represented as mean ± standard deviation of three independent experiments (*n* = 3). AMP, ampicillin; ERY, erythromycin; GA, gallic acid; HAMA, hamamelitannin; MIC, minimum inhibitory concentration.

The effects of the HAMA-ERY and GA-AMP combinations on the inhibition rates of bacteria over designated time intervals are shown in Figure 1. Compared with the untreated control, an almost 6-fold reduction in the cell density of *E. coli* ATCC 25922 was found after treatment for 24 h with hamamelitannin (1× MIC). In comparison with the drug-free control, >6-fold reduced density of *E. coli* cells was obtained after treatment for 24 h with ERY (1× MIC). The treatment of this bacterium with the combination of HAMA (½× MIC) + ERY (½× MIC) for 24 h exhibited an approximate 5-fold reduced growth compared with the drug-free control. Furthermore, nearly 4-fold growth of the bacterium was inhibited by the collective effects of both antibacterial agents (¼× MIC of both), which confirmed the promising antibacterial effect of this drug combination. Similarly, *E. coli* ATCC 25922 cells treated with GA (1× MIC) for 24 h exhibited an approximate 5-fold reduction in cell density compared with the untreated control. The treatment of *E. coli* cells with AMP (1× MIC) for 24 h showed an approximate 5-fold reduction in growth in comparison with the untreated control. Compared with the untreated control, the growth of this bacterium was inhibited by approximately 5-fold after treatment for 24 h with the GA (½× MIC) + AMP (½× MIC) combination.

### 2.4. Effects of Phenolic Compound-Antibiotic Combinations on Bacterial Cell Morphology

**Figure 2:**
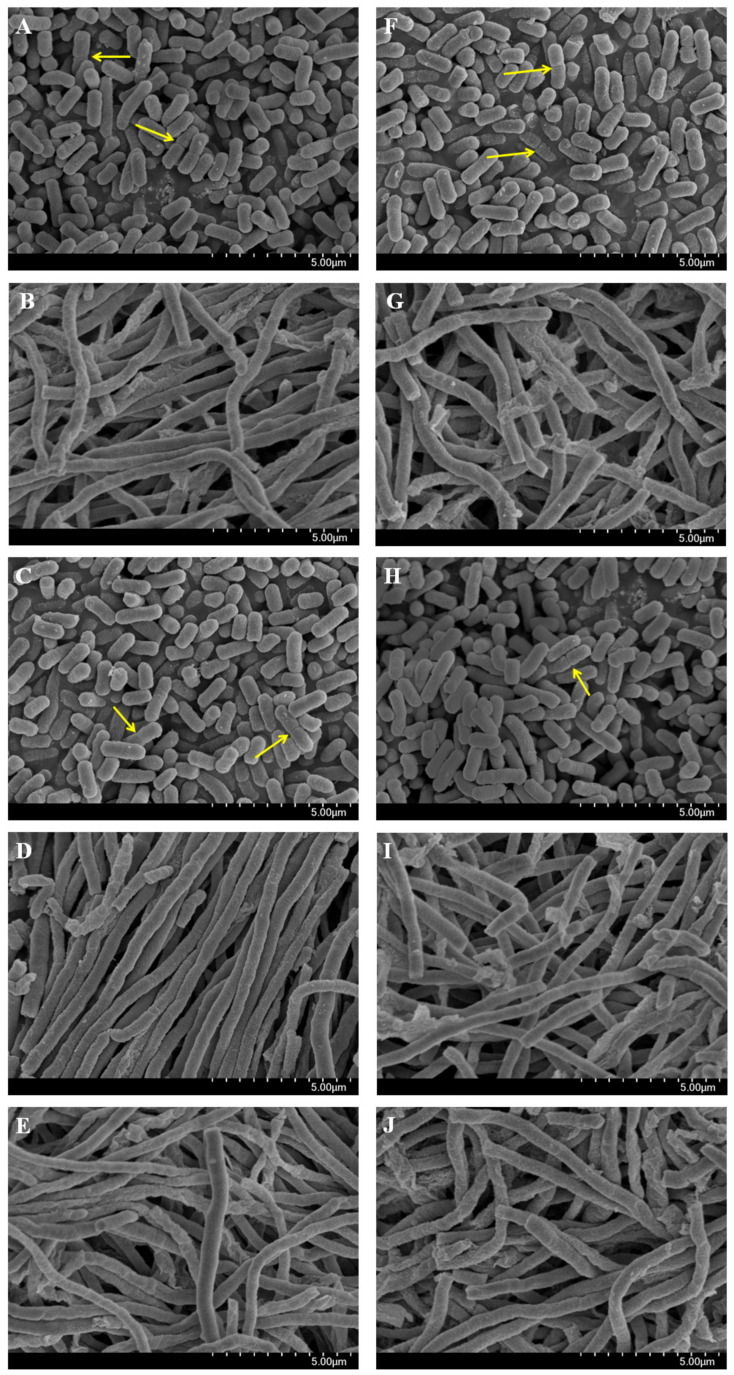
Effect of HAMA-ERY combination and GA-AMP combination on the ultrastructure morphology of *Escherichia coli* ATCC25922 cells. *Escherichia coli* cells treated with (**A**,**F**) no drug, (**B**) ERY (1× MIC), (**C**) HAMA (1× MIC), (**D**) HAMA (1× MIC) + ERY (1× MIC), (**E**) HAMA (½× MIC) + ERY (½× MIC), (**G**) AMP (1× MIC), (**H**) GA (1× MIC), and (**I**) GA (1× MIC) + AMP (1× MIC), (**J**) GA (½× MIC) + AMP (½× MIC), and examined the cells by scanning electronic microscope. Three independent experiments (*n* = 3) were performed. Representative images of bacterial cells treated with individual and combination antibacterials are shown, where the images are in 10,000× magnification. Yellow colored arrows indicate septa of binary fission. AMP, ampicillin; ERY, erythromycin; GA, gallic acid; HAMA, hamamelitannin; MIC, minimum inhibitory concentration.

To evaluate the effect of the HAMA-ERY and GA-AMP combinations on the cellular architecture, the morphology and ultrastructure of *Escherichia coli* ATCC 25922 cells treated with these antibacterial combinations were examined. Figure 2 presented the representative scanning electronic microscope (SEM) images of *E. coli* cells. As shown in the SEM images, the untreated (Figure 2A,F), HAMA (1× MIC)-treated (Figure 2C) and GA (1× MIC)-treated (Figure 2H) *E. coli* cells were found to be separated with perfect symmetry, and have a rod-like shape. Binary fissions of the bacterial cells were also found in these SEM images, which are indicated with yellow-colored arrows (Figure 2A,C,F,H). ERY (1× MIC)-treated (Figure 2B), HAMA (1× MIC) + ERY (1× MIC)-treated (Figure 2D), and HAMA (½× MIC) + ERY (½× MIC)-treated (Figure 2E) *E. coli* cells became rope-like, long, and without binary fissions, which was absolutely dissimilar to the untreated control cells. As found after the treatment of ERY (1× MIC), HAMA (1× MIC) + ERY (1× MIC), and HAMA (½× MIC) + ERY (½× MIC), similar morphology was obtained in AMP (1× MIC)-treated (Figure 2G), GA (1× MIC) + AMP (1× MIC)-treated (Figure 2I), and GA (½× MIC) + AMP (½× MIC)-treated (Figure 2J) *E. coli* cells.

### 2.5. Effects of Phenolic Compound-Antibiotic Combinations on the Inhibition and Viability of Biofilm

**Figure 3:**
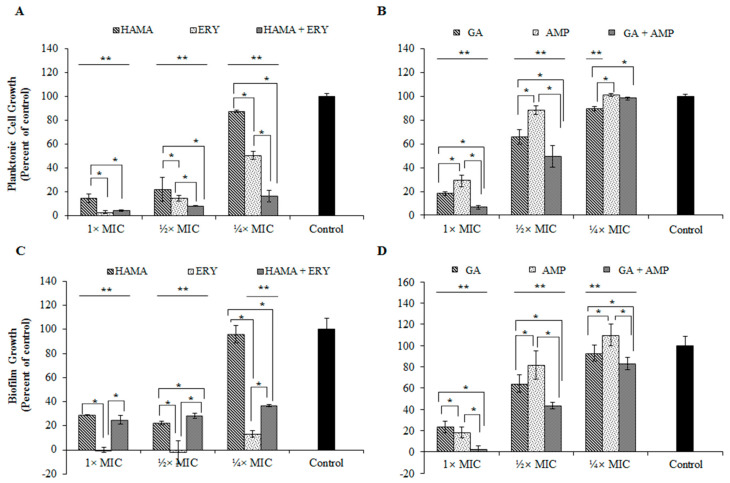
Effects of HAMA-ERY combination (**A**) and GA-AMP combination (**B**) on planktonic cells of *Escherichia coli* ATCC25922. Effects of HAMA-ERY combination (**C**) and GA-AMP combination (**D**) on biofilm cells of *Escherichia coli* ATCC25922. Data were analyzed by one-way analysis of variance with F-test. Data represented as mean ± standard deviation of three (*n* = 3) independent experiments. * Represents significance difference (*P* < 0.05) among the effects of individual drugs and combination drug within each concentration group. ** Represents significance difference (*P* < 0.05) compared with the untreated control group. AMP, ampicillin; ERY, erythromycin; GA, gallic acid; HAMA, hamamelitannin; MIC, minimum inhibitory concentration.

The formation of *E. coli* ATCC 25922 biofilms under the treatment of HAMA-ERY combination and GA-AMP combination were investigated. Figure 3 represented the effects of HAMA-ERY and GA-AMP combinations on the biofilm formation and planktonic cell growth in *E. coli*. The growth of the planktonic cells of *E. coli* was more extensively inhibited with the treatment of ERY (¼× MIC) in presence of HAMA (¼× MIC) (Figure 3A). The *E. coli* planktonic cell growth inhibition effect of ERY (½× MIC) was also significantly improved with the addition of HAMA (½× MIC). Similarly, the addition of GA (½× MIC) comprehensively increased the *E. coli* planktonic cell growth inhibition effect of AMP (½× MIC), and the supplementation of 1× MIC of GA in AMP (1× MIC) also showed same trend in inhibiting planktonic cells (Figure 3B). At ¼× MIC, AMP considerably hampered biofilm formation of *E. coli* when a lower concentration (¼× MIC) of GA was added with this antibiotic (Figure 3D). Addition of GA (½× MIC) to AMP (½× MIC), and the addition of GA (1× MIC) to AMP (1× MIC) extensively improved the biofilm inhibition effect of AMP (Figure 3D).

**Figure 4:**
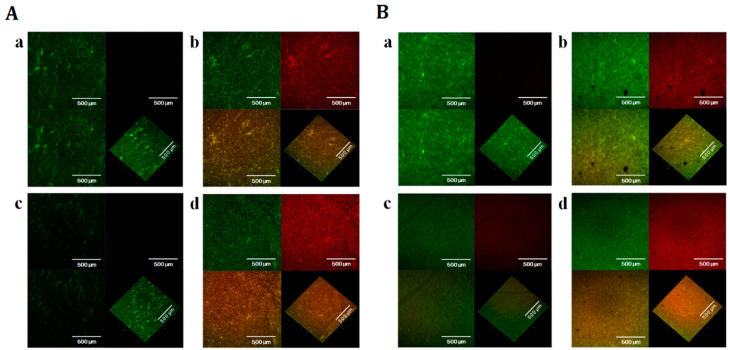
Effects of HAMA-ERY and GA-AMP combinations on the viability of *Escherichia coli* ATCC25922 biofilm. The CLSM images of SYTO9- and propidium iodide- stained biofilms after treated with (**A**) [(a) no drug, (b) HAMA (1× MIC), (c) ERY (1× MIC), (d) HAMA (1× MIC) + ERY (1× MIC)], and (**B**) [(a) no drug, (b) GA (1× MIC), (c) AMP (1× MIC), (d) GA (1× MIC) + AMP (1× MIC)]. The biofilm cell viabilities were evaluated by using BacLight LIVE/DEAD stain (red: dead cell, green: live cell). In each combined image such as image (a) or (b) or (c) or (d), the top-left image segment shows only the SYTO9-stained green fluorescent cells (live cells), the top-right image segment represents only the propidium iodide-stained red fluorescent cells (dead cells), and the below-left and -right image segments show both the SYTO9-stained green fluorescent cells (live cells) and propidium iodide-stained red fluorescent cells (dead cells) in together. In each combined image, all image segments are shown as two-dimensional except the below-right image segment; only the below-right image segment is represented as three-dimensional. Representative images of *Escherichia coli* biofilm cells from three independent experiments (*n* = 3) are presented in this figure. Images shown in this figure are in 50× magnification. AMP, ampicillin; CLSM, confocal laser scanning microscope; ERY, erythromycin; GA, gallic acid; HAMA, hamamelitannin; MIC, minimum inhibitory concentration.

**Figure 5:**
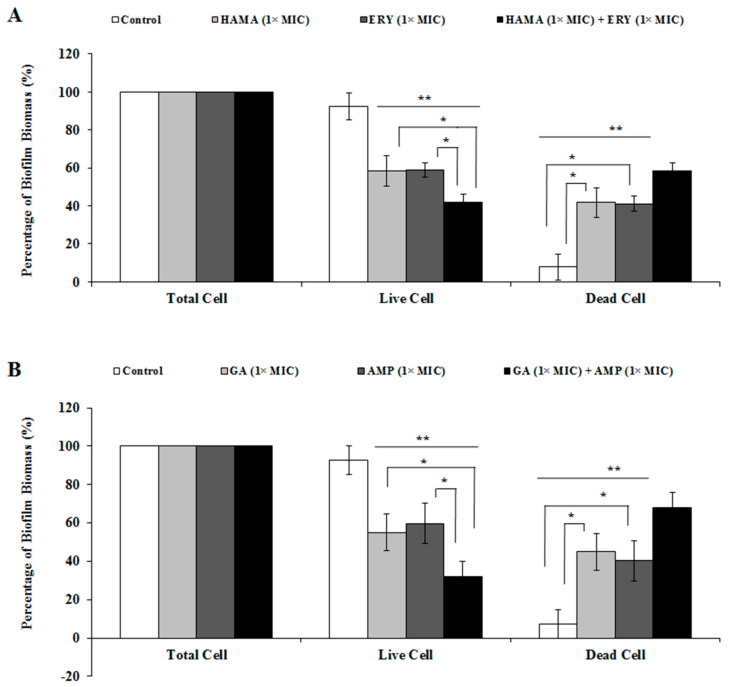
Percentages (%) of biofilm-biomasses of *Escherichia coli* after treatment with (**A**) HAMA-ERY and (**B**) GA-AMP combinations. In each test group, biomasses of “Total biofilm” were thought to be 100%, and with this consideration the percent (%) biomasses of dead (Propidium Iodide-stained) biofilm and live (SYTO9-stained) biofilm were calculated. Data were analyzed by one-way analysis of variance with F-test. Data represented as mean ± standard deviation of three (*n* = 3) independent experiments. * Represents significance difference (*P* < 0.05) among the effects of individual drugs and combination drugs within each concentration group. ** Represents significance difference (*P* < 0.05) compared with the untreated control group. AMP, ampicillin; ERY, erythromycin; GA, gallic acid; HAMA, hamamelitannin; MIC, minimum inhibitory concentration.

The effects of GA-AMP combination and HAMA-ERY combinations on the viability of *E. coli* biofilm cells were investigated by scanning the biofilm cells with confocal laser scanning microscope (CLSM) after staining the cells with BacLight live/dead stain. The reductions in viability of *E. coli* biofilm cells caused by the application of the HAMA-ERY and GA-AMP combinations were shown by the result attained with the CLSM. The images of *E. coli* biofilms obtained by CLSM are shown in Figure 4; the percentage biomass of *E. coli* biofilms that developed on the glass surfaces and when incubated in the presence of HAMA-ERY and GA-AMP combinations are presented in Figure 5. The CLSM-generated images of *E. coli* biofilm grown for 48 h and treated with HAMA-ERY and GA-AMP combinations for 24 h showed fewer ratios of live cells (green fluorescent cells) compared with the proportion in untreated controls. These images were analyzed by IMARIS software for determining the dead biofilm (fluorescent red cells) biomass and live biofilm (fluorescent green cells) biomass. The sum of the live biofilm biomass and dead biofilm biomass was considered as total biofilm biomass in a sample. It is clearly revealed from this result (Figure 5) that all biofilm cells in the untreated control sample were live, whereas a significant portion of biofilm cells were killed with the treatment of HAMA-ERY and GA-AMP combinations. The percentages of the biomasses of dead biofilm after treatment with the HAMA-ERY (58.27%) and GA-AMP (68.46%) combinations were noticeably higher than the percentages of the dead biofilm biomasses after treatment with the individual antibacterial agents (39.74–45.07%).

### 2.6. Effects of Phenolic Compound-Antibiotic Combinations on Bacterial Cell Motility

**Figure 6:**
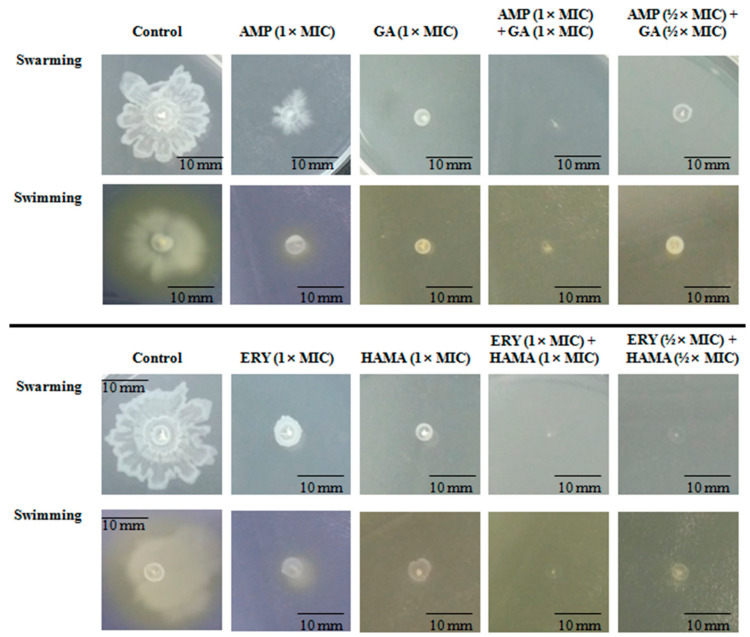
Representative images of swim and swarm zones of *Escherichia coli* ATCC25922 treated with HAMA-ERY and GA-AMP combinations. Representative images of three independent experiments (*n* = 3) are presented in this figure. Images shown in this figure are in 1× magnification. AMP, ampicillin; ERY, erythromycin; GA, gallic acid; HAMA, hamamelitannin; MIC: minimum inhibitory concentration.

The impacts of HAMA-ERY combination and GA-AMP combination on the swarming and swimming motilities of *E. coli* were assessed. Representative images of swarm cells and swim cells, which were either treated or untreated with combination antibacterial, are shown in Figure 6. The diameters of the swarm and swim zones are shown in Table 3. The swarm and swim motilities of *E. coli* were substantially restrained by the HAMA-ERY and GA-AMP combinations. Furthermore, the antibacterial combinations at sub-MIC concentration inhibited swarm and swim motilities of *E. coli* more intensely than those antibacterials individually could with their 1×MIC concentrations.

### 2.7. Effects of Phenolic Compound-Antibiotic Combinations on IEC-6 Cell Viability

**Figure 7:**
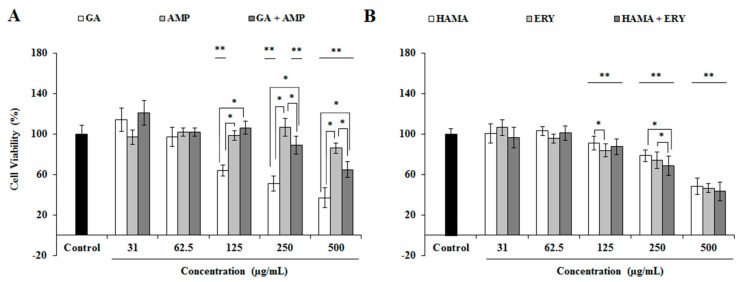
Effects of (**A**) HAMA-ERY and (**B**) GA-AMP combinations on the viability of *Rattus norvegicus* (IEC-6) cells. Data were analyzed by one-way analysis of variance with F-test. Data represented as mean ± standard deviation of three (*n* = 3) independent experiments. * Represents significance difference (*P* < 0.05) among the effects of individual drugs and combination drugs within each concentration group. ** Represents significance difference (*P* < 0.05) compared with the untreated control group. AMP, ampicillin; ERY, erythromycin; GA, gallic acid; HAMA, hamamelitannin.

The viabilities of small intestine cells (IEC-6) of *Rattus norvegicus* in presence of HAMA-ERY and GA-AMP combinations were studied. The rates of the viability of IEC-6 cells and the 50% inhibition concentration (IC_50_) of the tested antibacterial agents are shown in Figure 7 and Table S2. Individual treatment with HAMA (≤62.5 μg/mL) and GA (≤62.5 μg/mL) could not considerably affect the viabilities of the cells. Similarly, individual application of ERY and AMP up to concentrations of 62.5 and 250 μg/mL could not distress the viabilities of the cells. The GA-AMP combination (250 and 500 μg/mL) resulted in increased cell viability compared with the GA alone (Figure 7B). In contrast, the viabilities of the cells in presence of GA-AMP combination (250 and 500 μg/mL) were decreased in comparison with the viabilities found from the AMP alone. The viability rates of the cells after individual and combination treatment of HAMA and ERY were reduced compared to the untreated control. However, the viability of the cells after treatment with ERY or HAMA alone or with the combination was not significantly different. The IC_50_ values of HAMA and GA in IEC-6 cells were 988.54 μM and 564.55 μM, respectively. The addition AMP in GA solution increased the IC_50_ value of GA. Conversely, the supplementation of GA in AMP solution decreased the IC_50_ value of AMP. Moreover, the addition of HAMA (65–1032 µM) in ERY increased the IC_50_ value of ERY.

## 3. Discussion

Several recent reports indicate the formation of resistance by many bacterial strains to the diverse antibacterial drugs that have been widely utilized for treating the bacteria-associated infectious diseases in animal and humans [26]. Hence, there is an urgent need to develop alternative antibacterial agents able to combat resistant pathogen-associated infectious diseases [27,28]. The MIC values obtained in this study manifested the antibacterial effects of the tested phenolic-compounds against all these *E. coli* strains that were found to be resistant to between 2 and 10 of 10 traditional antibiotics (Table 1). The potential effects of the tested compounds were more obviously shown by their combination effects with traditional antibiotics, where they exhibited synergistic effects with ERY and thiamphenicol; either additive or indifferent interactions with the rest of the tested antibiotics against *E. coli*. It was also found that in the concentration- and time-dependent bacterial inhibition tests, the traditional antibiotics in combination with the phenolic compounds suppressed the growth of *E. coli* more potently than those antibiotics could inhibit individually. Moreover, the traditional antibiotics in combination with phenolic compounds demonstrated stronger inhibition of swarm motility, swim motility, and biofilm formation in *E. coli*.

The MIC of five phenolic compounds (GA, HAMA, epigallocatechin, epicatechin gallate, and epicatechin) was evaluated against five clinical strains and a QC strain of *E. coli*. GA has been reported to be effective in the suppression of the growth of many bacteria, including *P. aeruginosa*, *Salmonella typhi*, *E. coli*, and methicillin-sensitive and methicillin-resistant *Staphylococcus aureus* [29]. The MIC values obtained in this study (Table 1) showed that GA among the tested phenolic compounds enhanced the antibacterial activity the most, followed by epigallocatechin, HAMA, epicatechin gallate, and then epicatechin. Not only GA, but also epigallocatechin, exhibited very satisfying MIC values against *E. coli*. The MIC value of GA against *E. coli* in this study (1024 µg/mL) was lower than the MIC values (2500 µg/mL) reported previously [29]. The mean MIC value of plant-derived epigallocatechin against *E. coli* was reported as 733 ± 121 µg/mL, which was slightly lower and comparable with the results obtained in this study [30]. The MICs of epigallocatechin in resisting *E. coli* were lower in this study relative to the MICs of plant-extracted epigallocatechin in the previously published report. The variation in MICs of this compound in resisting *E. coli* may be due to the purity of the utilized epigallocatechin. Similarly, the MICs of epicatechin in resisting *E. coli* were 2500 µg/mL, which showed the similarity of the results obtained in our study with previously published results [29].

The treatment of bacterial infection with the combination of antimicrobial agents is suggested for preventing the development of antibiotic resistance and for improving the efficacies of antibacterial compounds as it is generally considered that the combination of antimicrobials is more efficacious when they possess synergistic effects; thus, it is a preferred technique [31]. As an example, the GA-thiamphenicol, HAMA-ERY, and HAMA-thiamphenicol combinations represented synergistic antibacterial effects in resisting *E. coli* with their FICI values of 0.281, 0.375, and 0.50, respectively. Moreover, additive antibacterial effects (FICI: 0.502–0.625) against *E. coli* were found from 17 combinations of antibacterial agents. Other antibacterial combinations in our study showed indifferent effects against *E. coli*. Outstanding in vitro antibacterial effects, together with the synergistic interactions with traditional antibiotics, underscore the potential appropriateness of these opportunistic molecules for treating the infections that are associated of *E. coli*. The combinations of HAMA-ERY and GA-AMP, which showed synergistic/additive effects in resisting *E. coli*, were carefully chosen for additional examinations based on their commercial and clinical significance.

The time-dependent killing assay is used to decide whether the effect of an antibacterial is bactericidal or bacteriostatic and to determine the pharmacodynamic features of new antimicrobial agents [32]. Bacteriostatic effects against the tested bacteria were observed in this study from GA and HAMA alone, and from the HAMA-ERY and GA-AMP combinations, as ≥99.9% reduction of the inoculum density was not found after 24 h of treatment in comparison with the control growth. Detectable growth of *E. coli* was not found at 12 h after treatment with the HAMA (1× MIC) and ERY (1× MIC) combination; beyond 12 h, the log phase had started and consequently reached its initial density within 24 h of treatment. Compared with the control, the density of bacterial cells was reduced by several-fold after treatment with GA or HAMA alone for 24 h, and the GA-AMP and HAMA-ERY combinations, which showed that the tested phenolic compounds, individually and together with ERY and AMP, exerted bacteriostatic effects.

Evaluation of the changes in the morphology, integrity, surface characteristics, and permeability of bacterial cell membrane yield important information regarding the mode of action of an antibacterial agent [33,34]. The morphological and physiological modifications in *E. coli* cells were determined by scanning the cells with SEM after treating the cells with HAMA or GA alone, and with the combinations of HAMA-ERY and GA-AMP. Both the HAMA-ERY and GA-AMP combinations resulted in direct effects on the morphology of the tested pathogen. In comparison with the untreated control cells, the treated *E. coli* cells had clear morphological variations. Bacterial cells treated with HAMA-ERY and GA-AMP combinations were revealed to be rope-like, long, and without binary fission, which was absolutely unlike the control cells. The results of this assay demonstrated that the antibacterial combinations had a vital impact on the cell division of *E. coli*. Similar effects were also found on *E. coli* cell morphology after the application of silver and gold nanoparticles [35]. The alteration in septum formation and the scarcity of the detachment of daughter cells at the time of cell division are two possible causes of this cell elongation effect [35].

It is reported that GA can inhibit the biofilm of *E. coli* and that HAMA has anti-biofilm effects against certain bacteria [36,37]. The effects of the HAMA-ERY and GA-AMP combinations on the formation of *E. coli* biofilms were determined in this study. In most cases, the antibacterial combinations more potently inhibited both the planktonic and biofilm cells of *E. coli* than the individual antibacterial agent could. The dead and live biofilm populations in presence of the combinations of HAMA-ERY and GA-AMP were ascertained by scanning the stained biofilm in CLSM. The enormous effects of the combination of GA-AMP on the *E. coli* biofilm cells was probably because of the smaller molecular weight of GA (170.12 g/mol), that can straightforwardly penetrate into the bio-films. Consequently, the antibacterial combinations appear to devastate the matrix of biofilm, which causes cell dissociation, and therefore the biofilm cells are further uncovered to the drugs and more vulnerable.

A pathogenic property of bacteria that is related to the dispersion and migration of bacterial cells is motility. Bacterial cells escape from the host immune response with the help of this pathogenic property [38]. The swimming motility is known to be driven by flagella. The flagella also functions in swarming motility, as well as in the formation of biofilm [30]. According to recently published reports, different types of antibiotics exhibit greater resistance to swarming cells than to biofilm cells [39,40]. The capability of the HAMA-ERY and GA-AMP combinations to inhibit the swarm and swim motilities of *E. coli* was evaluated in this study. The swarm motility and swim motility of *E. coli* were significantly inhibited by treatment with the HAMA-ERY and GA-AMP combinations (Figure 6), suggesting that these antibacterial combinations may have some impact on flagella-associated processes; specifically, flagella biosynthesis, chemotaxis, and rotation, which could lead to the decrease in swarming and swimming motilities.

In the study of the pharmacological effects of any drug, it is essential and desirable to determine the toxicity/safety profiles of that agent. In this study, the combinations of HAMA-ERY and GA-AMP were shown to have inhibitory effects against enteric pathogenic bacteria. If the ingested chemicals/toxins pass through the intestinal wall, then all tissues and organs are exposed to those chemicals/toxins in general. However, the highest concentration of ingested toxins/chemicals is first exposed and experienced by the gut among all organs. The functions of intestine in both humans and animals are affected by many of these toxins/chemicals [41]. Therefore, toxicological investigation focused on the intestine is of specific concern to this research. The impacts of HAMA and GA in IEC-6 cell viability were studied to determine any possible adverse effects of these compounds on intestinal cells. In IEC-6 cells, the IC_50_ values of HAMA and GA were 988.54 and 564.55 μM, correspondingly. The combination of AMP and GA at their highest tested concentrations (125–500 μg/mL) resulted in improved viability of IEC-6 cells compared with GA treatment alone. The combination of AMP with GA reduced the IC_50_ value of GA in IEC-6 cells. Likewise, the combination of HAMA (65–1032 µM) with ERY increased the IC_50_ value of ERY in IEC-6 cell. The impacts of HAMA and GA on the viability of various cell lines have been determined previously, and these compounds exerted no adverse effects or cytoprotective effects on cell viability [42,43]. Therefore, the effects of ERY-HAMA combination and AMP-GA combination on IEC-6 cells viability found in this study were reliable and logical.

Considering all the results of this investigation, we can conclude that HAMA-ERY and GA-AMP combinations are potent, promising, and novel agents for the eradication of pathogenic *E. coli.* The HAMA-ERY and GA-AMP combinations could more efficiently inhibit *E. coli* biofilm formation than ERY or AMP alone, which is crucial for the improvement of the efficacies of traditional antibiotics and/or the development of new antibacterials to minimize the pathogenic effects of *E. coli* as well as its impact on livestock and public health. Further study is suggested for confirming their safety and efficacy in in vivo systems to enhance their effective and safe utilization as medicines.

## 4. Materials and Methods 

### 4.1. Reagents, Chemicals, and Bacterial Strains

All reagents, chemicals, and microbiological culture media used in this study were from Sigma-Aldrich (St. Louis, MO, United States) unless otherwise mentioned. This study used a quality control strain ATCC 25922, and clinical strains V10-13-A02-002-008, V17-13-S02-002-009, V03-13-A01-002-003, V03-13-A03-002-009, and V16-13-S01-002-002 of *E. coli*, which were collected from different farms throughout the Republic of Korea. Clinical isolated strains V10-13-A02-002-008, V17-13-S02-002-009, V03-13-A01-002-003, V03-13-A03-002-009, and V16-13-S01-002-002 were from pig feces, chicken feces, cattle feces, chicken feces, and cattle carcass, respectively. Feces of animals were immediately collected after excretion in the farm. Similarly, carcass samples were instantly collected from freshly slaughtered cattle. Both kinds of specimens were immediately kept in iceboxes and then 10 g of each of these samples were transferred to sterile tubes. Nutrient broth (Kisan Bio Co., Ltd., Seoul, South Korea) with a volume of 45 mL were added to these tubes, and homogenized. One milliliter of these homogenates was transferred to a tube in 9 mL of buffered peptone water (Neogen, Lansing, MI, United States) and aerobically cultured for overnight at 37 °C. Then, 1 loop of cultures from each of these samples was streaked on eosin methylene blue agar (Kisan Bio Co., Ltd., Seoul, South Korea) and incubated for 24 h at 37 °C in aerobic condition. After incubating, the suspected colonies were biochemically identified as *E. coli* by using API 20E (bioMerieux Vitek Inc., Hazelwood, MO, United States). The isolated clinical strains were cultured for 20 h in Mueller Hinton broth (MHB; Becton Dickinson and Company, Becton Drive, NJ, United States) in a rotary incubator at 200 rpm and 37 °C before the experiments were performed.

### 4.2. Minimum Inhibition Concentrations of Phenolic Compounds and Conventional Antibiotics

The broth microdilution method of the CLSI was utilized to determine the MICs of the tested antibacterial agents [44]. Based on the CLSI guidelines, 5 × 10^5^ colony forming unit (CFU)/mL inoculum density in MHB was maintained for this assay. The solutions of the different antibacterial agents were diluted serially in 100 μL volumes in 96-well plates. The starting concentrations of HAMA, GA, epigallocatechin, epicatechin gallate, epicatechin, thiamphenicol, penicillin G, norfloxacin, marbofloxacin, florfenicol, ERY, ceftiofur, cefotaxime, AMP, and amoxicillin after the addition of diluted bacterial suspension in 96-well plates were 2048, 1024, 1024, 1024, 1024, 2048, 1024, 32, 32, 256, 2048, 256, 64, 1024, and 512 μg/mL, correspondingly, against all the tested strains. The suspensions were adjusted to 0.5 McFarland units by dilution of the cultures of different bacterial strains, and these cell density-adjusted diluted cultures were again diluted 100 times. Each of the antibiotic solutions (100 μL) was individually transferred to the wells of 96-well plate. The diluted suspensions of each of these *E. coli* strains with a volume of 100 μL were dispensed to the designated wells of these 96-well plates. The bacterial cells in different drug solutions were incubated for 18 h at 37 °C and the turbidity in all wells was analyzed. The lowest concentration of each antibacterial agent that fully prohibited any escalation in turbidity was determined as the MIC.

### 4.3. Fractional Inhibition Concentration (FIC) Index of Phenolic Compounds and Conventional Antibiotics

The combination effects of phenolic compounds and traditional antibiotics on *E. coli* ATCC 25922 were determined by the previously reported checkerboard microdilution method, with slight modifications [45]. For the comparison between phenolic compounds and traditional antibiotics, one was applied horizontally and the other one applied vertically in serial dilutions to obtain mixtures of these two antibacterial agents at various ratios in each well of a 96-well plate. The starting concentrations of these phenolic compounds and traditional antibiotics were the same as those used in the MIC determination described in the previous subsection. Similar dilutions of these antibacterial agents individually and the drug-free control wells (medium only) were also added in each test plate. The cultures of *E. coli* strains in their initial log phase were diluted, and these diluted suspensions of each of the *E. coli* strains with a volume of 100 μL were dispensed to each wells of the 96-well plates, where the final density of bacterial cells after dispensing to each well was 5 × 10^5^ CFU/mL. These bacterial cells in different drug solutions in 96-well plates were incubated at 37 °C for 18 h. The fractional inhibitory concentration (FIC) and the FICI were calculated from the following formulas [12].

(1) $$\mathrm{FIC}\;\mathrm{of}\;\mathrm{drug}\;A=\frac{\mathrm{MIC}\;\mathrm{of}\;\mathrm{drug}\;A\;\mathrm{in}\;\mathrm{presence}\;\mathrm{of}\;\mathrm{drug}\;B}{\mathrm{MIC}\;\mathrm{of}\;\mathrm{drug}\;A\;\mathrm{alone}}$$

(2) $$\mathrm{FIC}\;\mathrm{of}\;\mathrm{drug}\;B=\frac{\mathrm{MIC}\;\mathrm{of}\;\mathrm{drug}\;B\;\mathrm{in}\;\mathrm{presence}\;\mathrm{of}\;\mathrm{drug}\;A}{\mathrm{MIC}\;\mathrm{of}\;\mathrm{drug}\;B\;\mathrm{alone}}$$

FIC index = FIC of drug A + FIC of drug B. (3)

The effects of combinations of antibacterial agents were considered synergistic when the value of FICI was ≤0.5, additive when for 0.5 < FICI ≤ 1, indifferent when 1 < FICI ≤ 4, and antagonistic when FICI > 4 [46].

### 4.4. Effects of Phenolic Compound-Antibiotic Combinations on the Inhibition Rate of E. coli

Following the method of a previously reported article, the time- and concentration-dependent inhibitory effects of the HAMA-ERY and GA-AMP combinations on *E. coli* ATCC 25922 were investigated [12]. The inhibition rates of *E. coli* in presence of HAMA (1× MIC), ERY (1× MIC), HAMA (1× MIC) + ERY (1×MIC), HAMA (½× MIC) + ERY (½× MIC), and HAMA (¼× MIC) + ERY (¼× MIC) were investigated. Similarly, the effects of GA (1× MIC), AMP (1× MIC), GA (1× MIC) + AMP (1× MIC), GA (½× MIC) + AMP (½× MIC), and GA (¼× MIC) + AMP (¼× MIC) on the inhibition rates of *E. coli* were analyzed. An individual antibacterial agent or a combination of antibacterial agents was added to 10 mL MHB broth in a 15 mL Falcon tube. Cultures of *E. coli* in their initial log phase were diluted, and the diluted cultures were re-suspended in the drug-containing broth in the Falcon tube to a final bacterial cell density of 5 × 10^5^ CFU/mL. Similarly, a control tube of 10 mL MHB that contained the same bacterial cell density but had no antibacterial agent was prepared. Bacterial cells in drug-supplemented media were incubated in a shaking incubator at 200 rpm and 37 °C. One hundred microliters of the cultures were collected at the designated time points (0, 1, 2, 3, 4, 6, 8, 12, and 24 h) from each of these tubes, and then the cultures were serially diluted 10-fold in agar saline. Twenty microliter aliquots of the diluted cultures were spread on Mueller Hinton agar (MHA) in petri dishes and incubated overnight at 37 °C. The numbers of colonies in each of the diluted cultures were counted on the petri dishes to determine the CFUs of each culture. The mean log_10_ CFU/mL of each of these compounds was plotted for different time points.

### 4.5. Effects of Phenolic Compound-Antibiotic Combinations on the Morphology of E. coli Cells

The effects of the HAMA-ERY and GA-AMP combinations on the cell morphology of *E. coli* ATCC 25922 were estimated. Antibacterial solutions were added to MHB broth-containing 15 mL Falcon tubes where the volume of drug-supplemented MHB broth would be 10 mL and the final concentrations would be HAMA (1× MIC), ERY (1× MIC), HAMA (1× MIC) + ERY (1× MIC), HAMA (½× MIC) + ERY (½× MIC), AMP (1× MIC), GA (1× MIC), GA (1× MIC) + AMP (1× MIC), and GA (½× MIC) + AMP (½× MIC). Cultures of *E. coli* in their initial log phase were diluted and re-suspended in the drug-containing broth in a Falcon tube, to a final bacterial cell density of 5 × 10^5^ CFU/mL. Drug-free MHB broth (10 mL) in 15 mL Falcon tubes with the same bacterial density as the drug-containing broth was employed as the control. Bacterial cells in drug-supplemented medium were incubated in a shaking incubator at 200 rpm and 37 °C. In accordance with a reported procedure, the cells were collected, washed, and dried [47]. Scanning electron microscopes (models EDX-350 and S-4300; Hitachi, Japan) were used to determine the morphology of the ultrastructure of control- and drug-treated *E. coli* cells.

### 4.6. Effects of Phenolic Compound-Antibiotic Combinations on the Growth and Viability of E. coli Biofilm

A previously described spectrophotometric method was slightly modified and used to determine the effects of HAMA-ERY and GA-AMP combinations on the formation of biofilm by *E. coli* [48,49]. In brief, antibacterial agents were added to wells of a 96-well microplate where 100 μL of trypticase soy broth (TSB; Becton Dickinson and Company, Becton Drive, NJ, United States) were added earlier to each well of a 96-well microplate. Three separate wells were used for each concentration of antibacterial agent. The same antibacterial concentrations as stated in Section 4.4 were applied in this assay. The culture of *E. coli* in the initial log phase was diluted in TSB and re-suspended in the drug-containing broth in a 96-well microplate to a final bacterial cell density of 5 × 10^5^ CFU/mL after inoculation. Immediately after bacterial inoculation, the optical density (OD) value of the bacterial suspensions in the wells of a 96-well microplate were measured by using VersaMax microplate reader (Molecular Devices, San Jose, CA, United States) at a wavelength of 600 nm. The OD values of the bacterial suspensions in the wells of a 96-well microplate were measured again after incubation for 24 h at 37 °C to determine the planktonic cell growth. Subsequently, the supernatant of each well was carefully discarded without disturbing the biofilms that were attached to the inner surfaces of the well. The wells were washed three times in sterile phosphate buffered saline (PBS, pH 7.2) to remove the adherent drug and media components. Then, 200 μL of methanol (99%, *v*/*v*) was added to each well for 20 min to fix the biofilms. Crystal violet (100 µL; 0.2%, *w*/*v*) solution was added to the wells and kept for 15 min at room temperature to stain the biofilms. The wells were washed four times with PBS to remove the unbound or excess crystal violet. Then, 100 μL of ethanol (95%, *v*/*v*) was added to each of the wells to extract the crystal violet stain on the biofilm. The OD values of extracted crystal violet stain were measured and represented the comparative measurements of biofilm formation in comparison with the control.

The effects of the HAMA-ERY and GA-AMP combinations on the viability of the *E. coli* biofilms were evaluated in accordance with a previously reported biofilm viability assay [11,50]. Briefly, 2 mL of sterile TSB broth was dispensed to each of the chambers of a Nunc™ Lab-Tek™ II Chambered coverglass (ThermoFisher Scientific, Waltham, MA, USA). The culture of *E. coli* in its initial log phase was diluted in TSB and then inoculated into the broth of different chambers to a final bacterial cell density of 5 × 10^5^ CFU/mL after inoculation. The *E. coli* cells in TSB in different chambers were incubated for 48 h at 37 °C to allow biofilm formation. The broth medium used in the formation of biofilm was replaced by sterile and fresh medium every 24 h. After incubation for 48 h, the planktonic cells and the media were discarded, and 1× PBS was used to wash the chamber cover glasses. Then, 2 mL of HAMA (1× MIC), ERY (1× MIC), HAMA (1× MIC) + ERY (1× MIC), GA (1× MIC), AMP (1× MIC), and GA (1× MIC) + AMP (1× MIC) solutions in sterile TSB were individually added to different chambers and incubated for 24 h at 37 °C for the treatment of the developed biofilm cells. The biofilms treated for 24 h were washed with sterile double-distilled water (DDW). The washed biofilms were then stained with BacLight live/dead stain (ThermoFisher Scientific, Waltham, MA, United States). Biofilms that were not treated with any antibacterial agent were used as the control.

The BacLight live/dead staining kit contains propidium iodide and SYTO9 stains. We have described the principle of the BacLight live/dead staining assay in our previously published article [18]. In brief, the SYTO9 (green fluorescence)-stained biofilm cells are considered live cells owing to their membrane integrity. Conversely, the propidium iodide (red fluorescence)-stained cells are recognized as dead cells owing to their damaged membranes. A ZEISS LSM 700 CLSM (Carl Zeiss, Jena, Germany) was used for imaging. To image SYTO9-stained cells, an emission filter of 495–550 nm and a 488 nm laser were used, and for the scanning of propidium iodide-stained cells an emission filter of 560–600 nm and a 561 nm laser were used. The image acquisition and subsequent image handling were performed using ZEN 5.5 software (Carl Zeiss, Jena, Germany). Images were randomly taken from different parts of cover glass. The IMARIS 9.1 software package (Bitplane, Zurich, Switzerland) was used to analyze the images for quantification of the biomasses of live and dead biofilms. The sum of the live biofilm biomass and dead biofilm biomass was considered to represent the biomass of total biofilm.

### 4.7. Effects of Phenolic Compound-Antibiotic Combinations on Swim and Swarm Motilities of E. coli

The effects of phenolic compound-antibiotic combinations on the swim and swarm motilities of *E. coli* ATCC 25922 were investigated by slightly modifying a previously reported method [38,39]. Luria-Bertani (Becton Dickinson and Company, Becton Drive, NJ, United States) 0.8%, with 0.6% agar (Becton Dickinson and Company, Becton Drive, NJ, USA) and 0.5% glucose (Scharlab, Barcelona, Spain) was the composition of the medium used for the swarming assay of *E. coli*. The medium used for the evaluation of *E. coli* swim activity was composed of 1% Tryptone broth, 0.3% agar, and 0.5% NaCl (Scharlab, Barcelona, Spain). HAMA (1× MIC), ERY (1× MIC), HAMA (1× MIC) + ERY (1× MIC), HAMA (½× MIC) + ERY (½× MIC), GA (1× MIC), AMP (1× MIC), GA (1× MIC) + AMP (1× MIC), and GA (½× MIC) + AMP (½× MIC) were added in molten swimming/swarming medium in petri dishes. Negative control plates used for the swimming and swarming assay contained no antibacterial agents. The molten medium in petri dishes was kept inside a clean bench for 1 h to dry. Then, *E. coli* culture (2 μL) in its initial log phase was inoculated onto the swimming and swarming media in petri dishes. The *E. coli* cell-inoculated swarm and swim plates were kept at 37 °C in an incubator for 18 h and 10 h, respectively, for the incubation of motile cells. After the incubation period, the diameters of the swim and swarm zones were measured using calibrated digital slide callipers (Mitotoyo, Japan), and photographs of the swim and swarm zones were taken.

### 4.8. Effects of Phenolic Compound-Antibiotic Combinations on the Viability of Small Intestine Cells (IEC-6)

In accordance with the standard EZ-Cytox (EZ-1000; Daeillab Service Co. Ltd., Jeonju, South Korea) assay procedure, the effects of phenolic compound-antibiotic combinations on the viability of *Rattus norvegicus* small intestine cell line (IEC-6 cells; American Type Culture Collection CRL-1592, VA, United States) were determined in vitro. Briefly, IEC-6 cells were inoculated in Dulbecco’s Modified Eagle’s medium (DMEM; ThermoFisher Scientific, Waltham, MA, United States), supplemented with 4.5 g/L glucose, 1.5 g/L sodium bicarbonate (Carolina Biological Supply Company, Burlington, NC, United States), 4 mM L-glutamine (ThermoFisher Scientific, Waltham, MA, United States), and 0.1 units/mL fetal bovine serum (10%) and bovine insulin (90%). The cells were cultured in a humidified atmosphere at 37 °C with 5% carbon dioxide (CO_2_). Each week, the cells were passaged twice at a ratio of 1:5. The IEC-6 cell (2 × 10^4^ cells/mL) with a volume of 100 µL was suspended in the abovementioned DMEM in 96-well plates for 24 h at 5% CO_2_ and 37 °C. The medium from each well was aspirated and the cells were washed twice. Various concentrations of the antibacterial agents (100 µL) in the abovementioned DMEM were dispensed into each well. Then, IEC-6 cells in the presence of antibacterial agents in the abovementioned DMEM medium were incubated for 24 h at 5% CO_2_ and 37 °C. The EZ-Cytox dye (10 µL) was dispensed to each well and the IEC-6 cells were incubated again for 2 h. The OD of the cell suspension in each well was measured using a VersaMax microplate reader (Molecular Devices, San Jose, CA, United States) at a wavelength of 450 nm. Cells not treated with any drugs were assigned as the control. The cell viability (%) was calculated from the following formula where OD is the optical density [51]:

(4) $$\mathrm{Cell}\;\mathrm{viability}\;\left(\%\right)=\frac{\mathrm{OD}\;\mathrm{of}\;\mathrm{drug}-\mathrm{treated}\;\mathrm{sample}}{\mathrm{OD}\;\mathrm{of}\;\mathrm{untreated}\;\mathrm{sample}}\times 100$$

### 4.9. Statistical Analysis

The results are presented as the mean ± standard deviation (SD) of the triplicate analysis. Statistical analysis was performed using SAS software (SAS Institute Inc., Cary, NC, USA). One-way analysis of variance (ANOVA) followed by F-test was used to compare the results. Statistical significance was considered for *P*-values of < 0.05.

## Figures and Tables

**Table 1:** Minimum Inhibition Concentration (MIC, µg/mL) of commercially available antibiotics and opportunistic antibacterial agents against some clinically isolated strains of *Escherichia coli*, and the sensitivity status of those isolated *Escherichia coli* strains to those traditional antibiotics.

Traditional Antibiotics/Antibacterial Agents	ATCC 25922	V10-13-A02-002-008	V17-13-S02-002-009	V03-13-A01-002-003	V03-13-A03-002-009	V16-13-S01-002-002
		Pig Feces	Chicken Feces	Cattle Feces	Chicken Feces	Cattle Carcass
AMX	1 (S)	256 (R)	128 (R)	16 (R)	256 (R)	4 (S)
AMP	2 (S)	512 (R)	256 (R)	64 (R)	512 (R)	8 (S)
CEF	1 (S)	128 (R)	64 (R)	1 (S)	64 (R)	1 (I)
CEFO	0.125 (S)	64 (R)	32 (R)	1 (S)	32 (R)	1 (S)
ERY	128 (R)	512 (R)	1024 (R)	16 (R)	512 (R)	64 (R)
FLOR	8 (I)	64 (R)	64 (R)	2 (S)	32 (R)	1 (S)
NOR	0.5 (S)	16 (R)	4 (R)	0.50 (S)	8 (R)	0.50 (S)
MAR	0.25 (S)	1 (I)	2 (R)	0.50 (S)	1 (I)	0.25 (S)
THIA	256 (R)	512 (R)	512 (R)	128 (R)	512 (R)	256 (R)
PENG	16 (I)	1024 (R)	256 (R)	32 (R)	>1024 (R)	32 (R)
EPI	>1024	>1024	>1024	>1024	>1024	>1024
EPIG	1024	512	512	256	512	512
EPGC	512	1024	512	512	512	512
GA	1024	1024	1024	256	512	256
HAMA	2048	512	1024	1024	1024	1024

Antibacterial sensitivity status of these clinically isolated *Escherichia coli* strains were determined by interpreting the MIC break-point values of these traditional antibiotics reported by various organizations and the MIC results of these traditional antibiotics obtained in this study against those isolated *Escherichia coli* strains [19,20,21,22,23,24,25]. Three independent experiments (*n* = 3) were performed. In each experiment triplicate samples were assayed for each sample group. AMP, ampicillin; AMX, amoxicillin; CEF, ceftiofur; CEFO, cefotaxime; EPGC, epigallocatechin; EPI, epicatechin; EPIG, epicatechin gallate; ERY, erythromycin; FLOR, florfenicol; GA, gallic acid; HAMA, hamamelitannin; I, intermediate resistance; MAR, marbofloxacin; NOR, norfloxacin; PENG, penicillin G; R, resistant; S, susceptible; THIA, thiamphenicol.

**Table 2:** Effects of phenolic compound-antibiotic combinations on the inhibition of *Escherichia coli* ATCC25922.

Drug Combinations	FICI
GA + AMP	0.504 (A)
GA + Amoxicillin	1.008 (I)
GA + Ceftiofur	1.001 (I)
GA + Penicillin G	1.004 (I)
GA + Cefotaxime	0.563 (A)
GA + ERY	1.002 (I)
GA + Thiamphenicol	0.281 (S)
GA + Marbofloxacin	0.625 (A)
HAMA + AMP	1.001 (I)
HAMA + Amoxicillin	0.625 (A)
HAMA + Ceftiofur	1.002 (I)
HAMA + Penicillin G	1.002 (I)
HAMA + Cefotaxime	1.001 (I)
HAMA + ERY	0.375 (S)
HAMA + Thiamphenicol	0.500 (S)
HAMA + Marbofloxacin	0.625 (A)
Epicatechin + AMP	1.004 (I)
Epicatechin + Amoxicillin	1.001 (I)
Epicatechin + Ceftiofur	0.625 (A)
Epicatechin + Penicillin G	1.002 (I)
Epicatechin + Cefotaxime	0.563 (A)
Epicatechin + ERY	0.504 (A)
Epicatechin + Thiamphenicol	1.008 (I)
Epicatechin + Marbofloxacin	0.504 (A)
AMP + Epicatechin gallate	1.004 (I)
Amoxicillin + Epicatechin gallate	1.001 (I)
Ceftiofur + Epicatechin gallate	0.625 (A)
Penicillin-G + Epicatechin gallate	1.002 (I)
Cefotaxime + Epicatechin gallate	1.002 (I)
ERY + Epicatechin gallate	1.001 (I)
Thiamphenicol + Epicatechin gallate	0.625 (A)
Marbofloxacin + Epicatechin gallate	0.516 (A)
Epigallocatechin + AMP	0.504 (A)
Epigallocatechin + Amoxicillin	1.008 (I)
Epigallocatechin + Ceftiofur	0.625 (A)
Epigallocatechin + Penicillin G	1.008 (I)
Epigallocatechin + Cefotaxime	0.502 (A)
Epigallocatechin + ERY	0.516 (A)
Epigallocatechin + Thiamphenicol	1.008 (I)
Epigallocatechin + Marbofloxacin	0.504 (A)

Five independent experiments (*n* = 5) were performed. A, additive; AMP, ampicillin; ERY, erythromycin; FICI, fractional inhibitory concentration index; GA, gallic acid; HAMA, hamamelitannin; I, indifferent; S, synergy; Synergy, FICI ≤ 0.5; Additive, 0.5 < FICI ≤ 1; Indifferent, 1 < FICI ≤ 4; Antagonist, FICI > 4.

**Table 3:** Effects of HAMA-ERY combination and GA-AMP combination on swarm motility and swim motility of *Escherichia coli*.

Test Groups	Swarm Zone Diameter (mm) (mean ± SD)	Swim Zone Diameter (mm) (mean ± SD)
Control	23.33 ± 2.08	22.00 ± 1.73
ERY (1× MIC)	6.67 ± 1.15 ***^,#^	5.67 ± 0.58 **^,#^
HAMA (1× MIC)	3.33 ± 0.58 **^,#^	7.33 ± 1.53 ***^,#^
HAMA (1× MIC) + ERY (1× MIC)	0.00 ± 0.00 *^,#^	0.00 ± 0.00 *^,#^
HAMA (½× MIC) + ERY (½× MIC)	0.00 ± 0.00 *^,#^	2.67 ± 0.58 *^,#^
Control	21.67 ± 2.52	21.33 ± 3.06
AMP (1× MIC)	11.33 ± 1.53 ***^,#^	5.67 ± 1.15 ***^,#^
GA (1× MIC)	4.00 ± 1.73 **^,#^	4.33 ± 1.53 **^,#^
GA (1× MIC) + AMP (1× MIC)	0.00 ± 0.00 *^,#^	1.67 ± 1.53 *^,#^
GA (½× MIC) + AMP (½× MIC)	3.67 ± 1.15 **^,#^	4.00 ± 1.00 **^,#^

Data were analyzed by one-way analysis of variance with F-test. Data represented as mean ± standard deviation of three (n = 3) independent experiments. *, ** and *** represent significance differences among the effects of individual drugs and combination drug with the *P*-value of (*P* < 0.05), (*P* < 0.001), respectively. ^#^ Represents significance difference (*P* < 0.05) compared with the untreated control group. AMP, ampicillin; ERY, erythromycin; GA, gallic acid; HAMA, hamamelitannin; MIC: minimum inhibitory concentration; SD, standard deviation.

## Supplementary Materials

The following are available online at https://www.mdpi.com/2076-0817/9/10/811/s1. Supplementary information includes one table: Table S1: Effects of hamamelitannin-erythromycin and gallic acid-ampicillin combinations on the viability of *Rattus norvegicus* small intestine (IEC-6) cells.
