# Supplementary material for: Synergism of the Combination of Traditional Antibiotics and Novel Phenolic Compounds against Escherichia coli
Source: Pathogens. 2020 Oct 3;9(10):811. doi: 10.3390/pathogens9100811 (PMC7600547; doi:10.3390/pathogens9100811)
Supplement: Supplementary file 1 [file pathogens-09-00811-s001.pdf]

Table S1: Effects of hamamelitannin-erythromycin and gallic acid-ampicillin combinations on the viability of *Rattus norvegicus* small intestine (IEC-6) cells.

**Antibacterials**	**Inhibitory Concentration 50% (µM)**
Ampicillin	2556.66
Erythromycin	515.50
Gallic acid	564.55
Hamamelitannin	988.54
Gallic acid + (89-1431) µM Ampicillin	1746.86
Ampicillin + (184-2939) µM Gallic acid	828.90
Hamamelitannin + (43-681) µM Erythromycin	564.55
Erythromycin + (65-1032) µM Hamamelitannin	777.09

Results are interpreted from 3 independent experiments.
